# Editorial: Deep Learning for Toxicity and Disease Prediction

**DOI:** 10.3389/fgene.2020.00175

**Published:** 2020-02-26

**Authors:** Ping Gong, Chaoyang Zhang, Minjun Chen

**Affiliations:** ^1^Environmental Laboratory, U.S. Army Engineer Research and Development Center, Vicksburg, MS, United States; ^2^School of Computing Sciences and Computer Engineering, University of Southern Mississippi, Hattiesburg, MS, United States; ^3^Division of Bioinformatics and Biostatistics, National Center for Toxicological Research, U.S. Food and Drug Administration, Jefferson, AR, United States

**Keywords:** deep learning, disease diagnosis or prognosis, chemical toxicity prediction, deep neural networks, conventional machine learning

Deep learning (DL), alsocalled deep structured learning or hierarchical learning, is an important subset of machine learning (ML). The distinction between DL and conventional “shallow” ML is that DL algorithms allow computational models composed of multiple processing layers to be fed with raw data and automatically learn multiple levels of abstract representations of data for detection and classification (LeCun et al., 2015). The history of DL can be traced back to the 1940s when the first neural network model was developed (McCulloch and Pitts, 1943). It wasn't until recently that DL evolved into and reemerged as a prominent discipline within the artificial intelligence domain, thanks to such revolutionary advances as backpropagation, parallel computing with GPUs, availability of massive labeled data, improved architectures, robust optimizers, regularization techniques, and activation functions (see https://www.import.io/post/history-of-deep-learning/ and https://beamandrew.github.io/deeplearning/2017/02/23/deep_learning_101_part1.html for more info). Over the past decade DL has regained popularity and has been successfully applied to such diverse fields as image (Zeiler and Fergus, 2014) and speech (Hinton et al., 2012) recognition, visual art (Huang et al., 2016) and natural language (Xiong et al., 2016) processing, drug discovery (Gawehn et al., 2016), chemical toxicity prediction (Mayr et al., 2016), and computational biology (Angermueller et al., 2016). For instance, deep convolutional neural networks (CNNs) have brought about breakthroughs in computer vision and pattern recognition (Krizhevsky et al., 2012), whereas recurrent neural networks have shed light on sequential data such as text mining and speech applications (Hinton et al., 2012).

Despite great success, there remain many technical challenges, one of which is how to integrate or transform subject-specific knowledge in order to adapt to DL algorithms and improve outcomes. Technical hurdles exist in data preprocessing, model selection (e.g., feedforward, convolutional, or recurrent networks), parametric function approximation (e.g., initialization strategies, activation functions, architecture, and learning techniques), and model regularization and optimization. This Research Topic addresses these challenges and hurdles with a specific focus on the application of DL algorithms to chemical toxicity prediction and disease diagnosis, which has not been adequately explored (Mayr et al., 2018; Xu et al., 2019). As a result, 11 manuscripts were accepted in four participating journals: 7 in Frontiers in Genetics (Zhang L. et al.; Hu et al.; Jia et al.; Luo et al.; Xie et al.; Zhang X. et al.; Ji et al.), 2 in Frontiers in Plant Science (Fuentes et al.; Lin et al.), 1 in Frontiers in Physiology (Idakwo et al.), and 1 in Frontiers in Bioengineering and Biotechnology (Matsuzaka and Uesawa). These papers are well-split between human (Zhang L. et al.; Jia et al.; Luo et al.; Xie et al.; Zhang X. et al.) or plant (Fuentes et al.; Lin et al.) disease diagnosis and chemical toxicity (Matsuzaka and Uesawa; Idakwo et al.) or drug efficacy (Hu et al.; Ji et al.) prediction. CNN architecture dominated these studies, except three where autoencoder (Zhang L. et al.; Hu et al.) or XGBoost (Ji et al.) was employed. The input data varied from images (Fuentes et al.; Lin et al.; Xie et al.) or converted images (Matsuzaka and Uesawa) to gene mutations (Luo et al.), chemical molecular descriptors (Hu et al.; Idakwo et al.), phenotypes (Jia et al.), physical examination records (Zhang X. et al.), and mixtures of different data profiles such as multi-omics data (Zhang L. et al.), chemical structures, human phenotypes, pathways, protein targets, and protein–protein interactions (Ji et al.).

As summarized below, this collection of original research papers presents a significant amount of progress made in the above-mentioned scope of the Research Topic:

**Development of novel DL-based tools:** Autoencoder-based classification models were developed to identify ultra-high risk prognostic subgroups of neuroblastoma (Zhang et al.) or distinguish drug-like compounds from common compounds (Hu et al.). Luo et al. demonstrated that a CNN-based deepDriver could learn information within somatic mutation data and similarity networks simultaneously to enhance the prediction of cancer driver genes. A CNN-based, pixel-level semantic segmentation model was built for quantitative assessment of the severity of powdery mildew in cucumber leaves, achieving an average pixel accuracy of 96% (Lin et al.). Xie et al. applied both CNN- and autoencoder-based DL and transfer learning techniques to automatically extract high-level abstract features from breast cancer histopathological images, which led to a significant improvement in cancer diagnosis. Zhang X. et al. reported a novel GroupNet model for multi-label chronic disease classification that outperformed other DL (e.g., AlexNet) and conventional ML (e.g., SVM) models.

**Optimization of existing DL-based tools:** Fuentes et al. presented a two-tiered diagnosis system to address high false positive rates caused by class unbalance and variation. The system consists of a primary diagnosis unit that detects a set of bounding boxes that likely contain a disease in the image, a secondary diagnosis unit that verifies bounding boxes detected from the primary diagnosis unit using independent CNN classifiers trained with respect to each class, and an integration unit that combines the results from the primary and secondary units to effectively recognize 10 different types of diseases and pests in tomato. This system showed an improved recognition rate of 96%, 13% higher than previous work (Fuentes et al., 2017). Matsuzaka and Uesawa refined DeepSnap, a DL-based tool for quantitative structure-activity relationship (QSAR) analysis previously developed by Uesawa (2018), through optimizing such parameters as the number of molecules per Structure Data File (SDF), zoom factor percentage, atom size for van der Waals percentage, bond radius, minimum bond distance, and bond tolerance. The DeepSnap with an optimal set of parameter values generated the best performing models.

**Choosing between DL and conventional ML (cML):** Despite revolutionary breakthroughs, DL does not always provide better performance or superior solutions to any specific problem than cML. Such cML as Logistic Regression (LR), Random Forest (RF), and Naive Bayes (NB) were employed along with Deep Neural Network (DNN) to train classifiers with excellent precision (≥98%) and recall (up to 95%) for rare disease diagnosis implemented in a Rare Disease Auxiliary Diagnosis system (Jia et al.). Idakwo et al. presented a case study where DNN and RF were compared with and without parametric optimization in terms of QSAR-based chemical toxicity prediction. Ji et al. compared XGBoost, a cML algorithm, with DeepSynergy, a DL algorithm, and other cML algorithms (e.g., RF, LR, and NB), and concluded that XGBoost outperformed other classifiers in both stratified five-fold cross-validation and independent validation in identifying synergistic or antagonistic drug combinations. These studies suggest that in the absence of large amounts of training samples (e.g., in the 100 or 1,000 k range), cML may be an alternative superior to DL in performance, as cML is less likely to over-fit and often computationally less costly. Even with available big data, DL algorithms need to be optimized to achieve outstanding performance (Fuentes et al.; Idakwo et al.; Matsuzaka and Uesawa). Furthermore, transfer learning was used in conjunction with DL to train a neural network model on a problem similar to the one being solved (Xie et al.; Matsuzaka and Uesawa).

**Data preprocessing:** In order to take advantage of the power of CNN, Matsuzaka and Uesawa converted SMILES text files into SDF image files, whereas Zhang X. et al. transformed physical examination records into multi-label class data using binary relevance and label powerset methods. Data rebalance techniques (Hu et al.; Xie et al.) and focal loss (Zhang X. et al.) or stratification (Ji et al.; Idakwo et al.) strategies were often performed to overcome the influence of skewed class distribution. Data preprocessing played a critical role in improving performance of DL- or cML-based classification.

This collection of contributions highlights not only the promising outlook of DL applications in disease diagnosis and toxicity prediction, but also the necessity of optimizing DL algorithms in order to achieve superior outcomes. Given the remarkable success of DL application in classification problems, the focus of future efforts may now shift to quantification problems.

## Author Contributions

PG proposed and edited this Research Topic. CZ and MC co-edited this Research Topic. All authors made a substantial, direct and intellectual contribution to this Editorial, and approved it for publication.

### Conflict of Interest

The authors declare that the research was conducted in the absence of any commercial or financial relationships that could be construed as a potential conflict of interest.
